# Supplementary material for: Polycation-Intercalated MXene Membrane with Enhanced Permselective and Anti-Microbial Properties
Source: Nanomaterials (Basel). 2023 Oct 31;13(21):2885. doi: 10.3390/nano13212885 (PMC10650023; doi:10.3390/nano13212885)
Supplement: Supplementary file 1 [file nanomaterials-13-02885-s001.zip › nanomaterials-2657517-supplementary.pdf]

Polycation-Intercalated MXene Membrane with Enhanced Permselective and Anti-Microbial Properties

Jie Yang [1,*], Shilin Zhu [2] and Hongli Zhang [2,*]

[1] School of Materials Science and Engineering, Xi'an Polytechnic University, Xi'an 710048, China
[2] School of Materials Science and Chemical Engineering, Xi'an Technological University, Xi'an 710021, China; shilin971020@163.com
* Correspondence: jieyang0320@126.com (J.Y.); zhanghongli@xatu.edu.cn (H.Z.)

Supplementary Figures

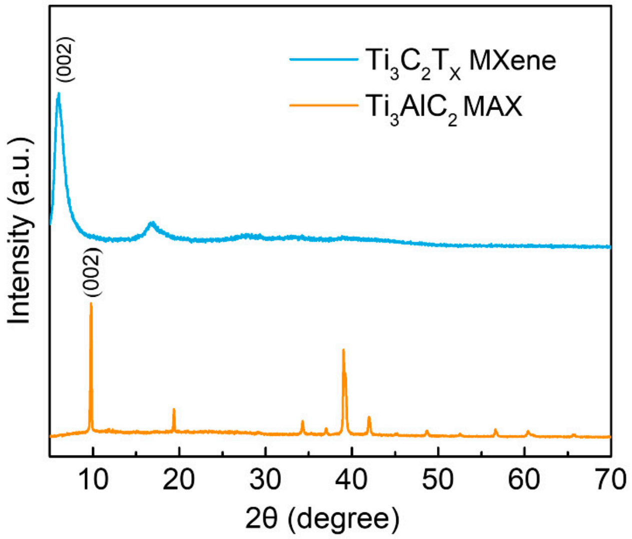

Figure S1 (a) XRD pattern of the Ti_3AlC_2 MAX powder and $Ti_3C_2T_x$ MXene.

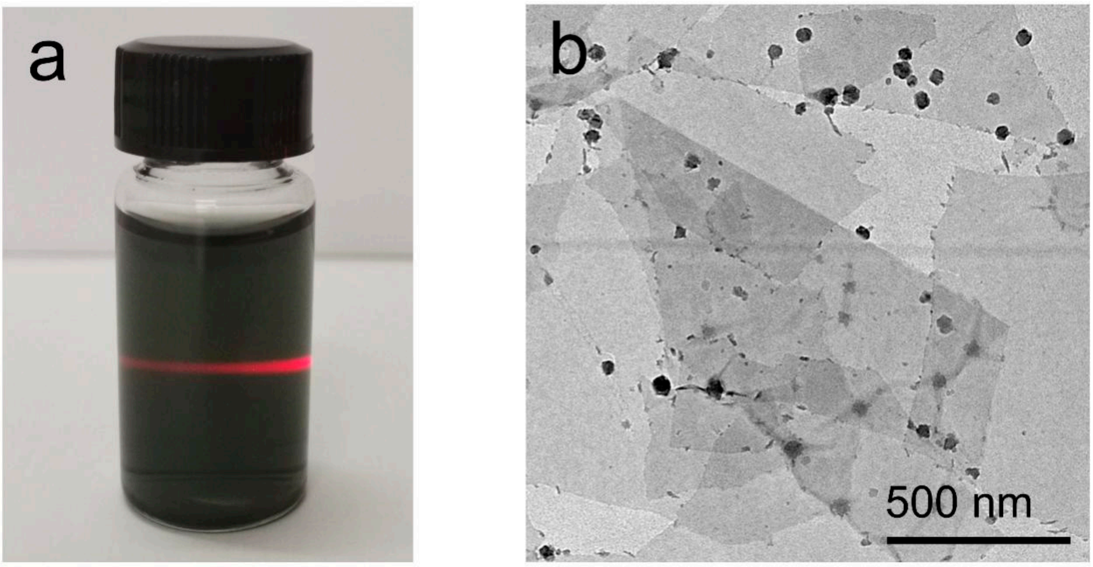

Figure S2 (a) Photograph of few-layerd $Ti_3C_2T_x$ MXene suspension with a significant Tyndall scattering effect, (b) TEM image of the $Ti_3C_2T_x$ MXene nanosheets.

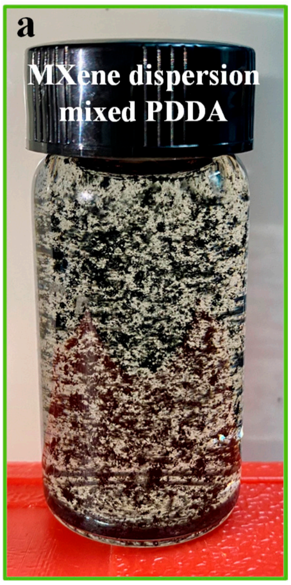

Figure S3 Mixed MXene dispersion and PDDA solution

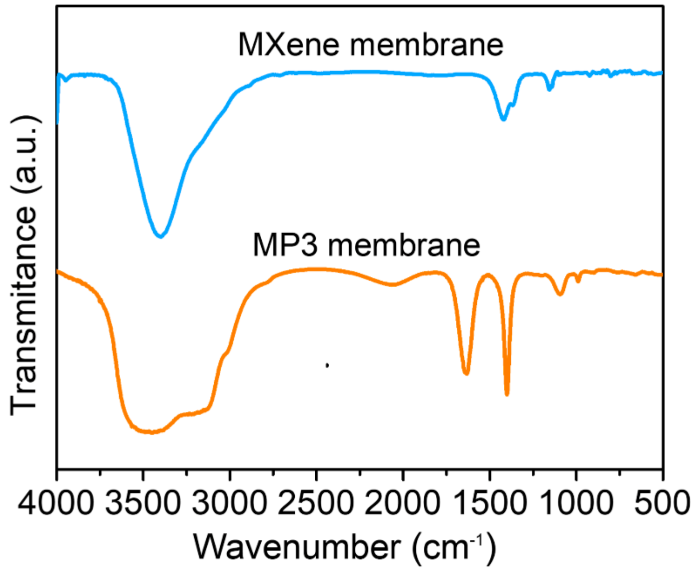

Figure S4 FTIR of the MXene membrane and MP3 membrane.

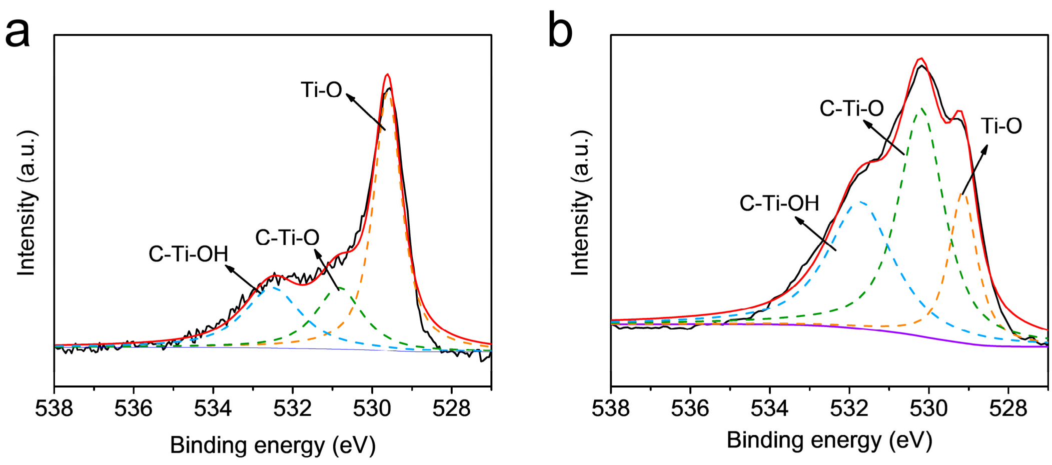

Figure S5 High-resolution O1s XPS spectra of (a) MXene membrane and (b) MXene/PDDA membrane.

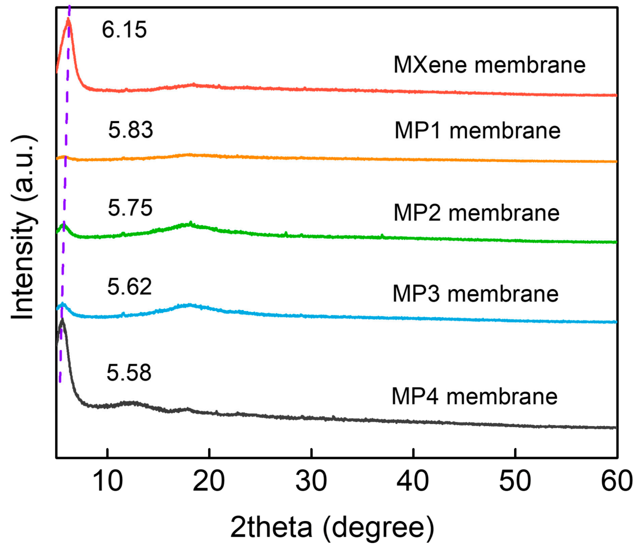

Figure S6 XRD patterns of the MXene/PDDA membrane with different PDDA content.

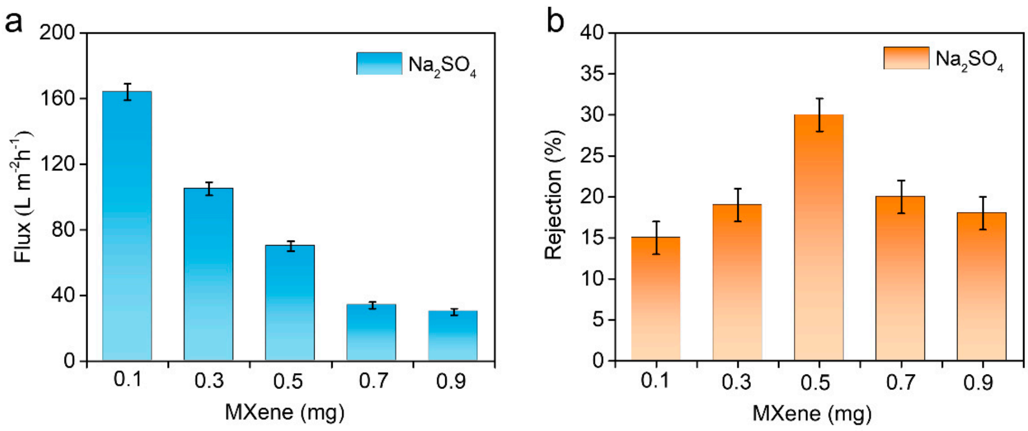

Figure S7 Separation performance of pure MXene membrane with different thickness.

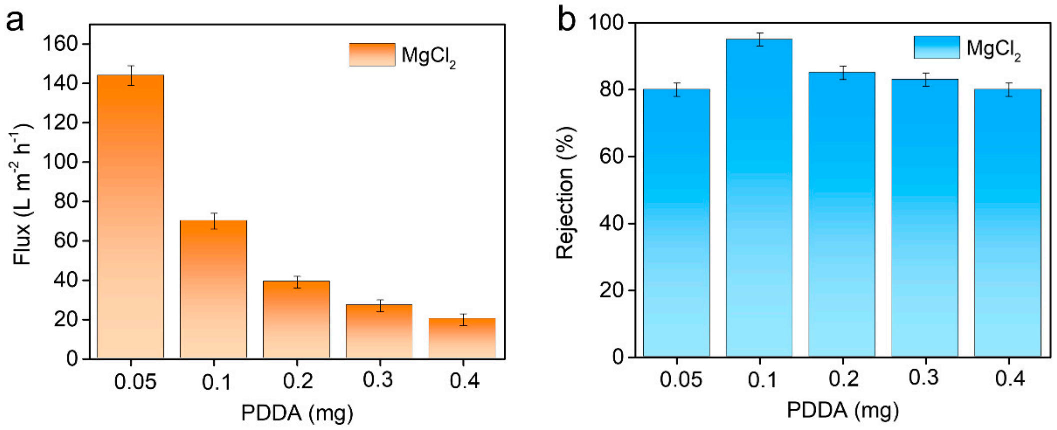

Figure S8 Separation performance of $Ti_3C_2T_x$/PDDA membrane with different PDDA content.

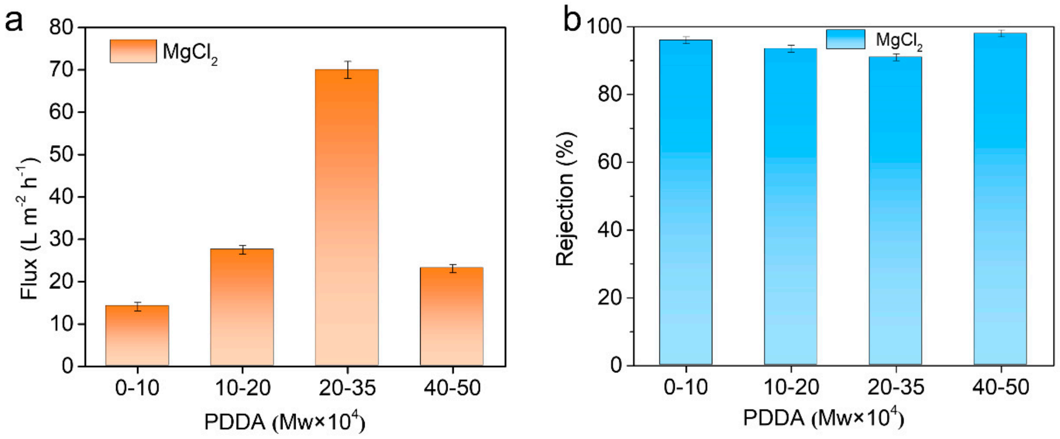

Figure S9 Separation performance of $Ti_3C_2T_x$/PDDA membrane with different molecular weight of PDDA.

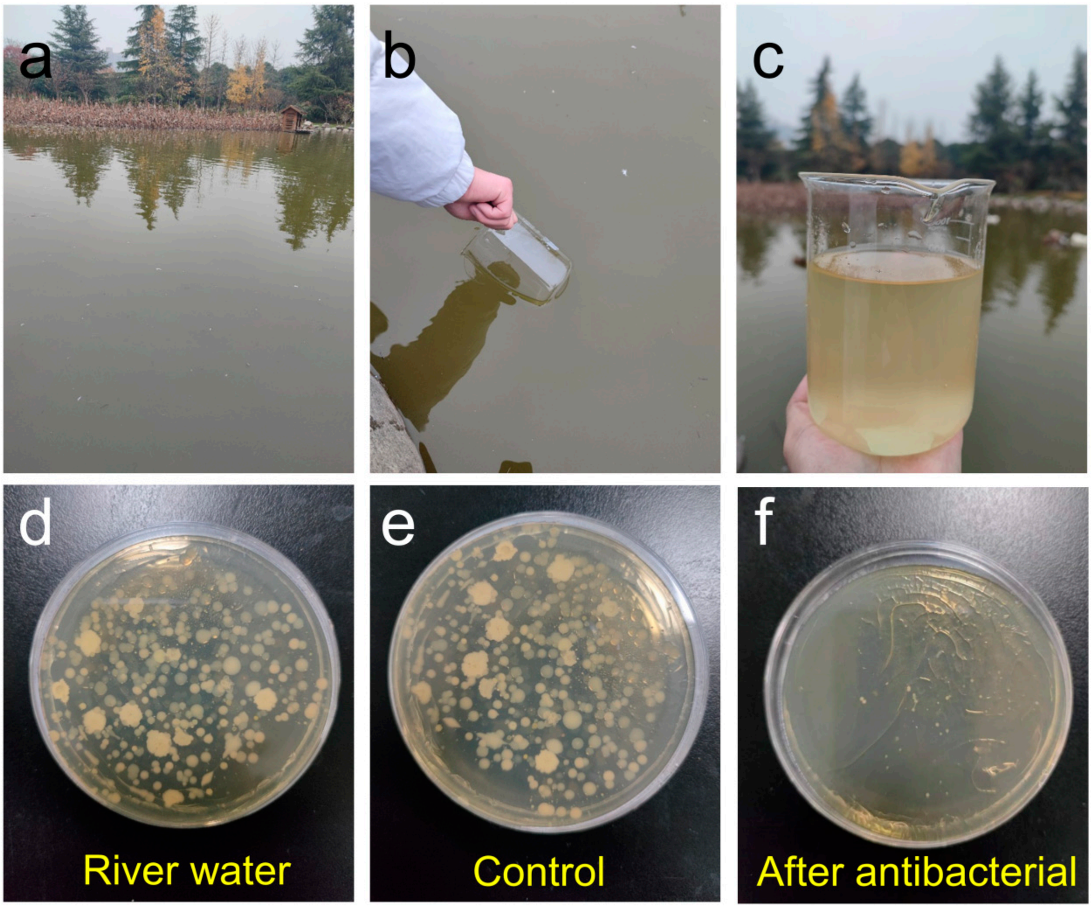

Figure S10 (a-c) Collection of the waste water from river; (d-f) Practical antibacterial results of the MP3 membrane.

Table S1 Comparison of membranes investigated in this work with other two-dimensional membranes in literature.

Membrane	Water flux (L $m^{-2} \cdot h^{-1}$)	Rejection (%)	FRR (%)	Antibacterial activity	References
MP3	73.4	94.6%($MgCl_2$)	96.1	*E. coli*: 90<br>*S. aureus*: 95	*This work*
MXene	/	/	/	*E. coli*: 67<br>*B. subtilis*: 73	[43]
MXene@CA	256.85	100（BSA）	/	*E. coli*: 98<br>*B. subtilis*: 96	[21]
AgCl-TiO_2 membrane	/	/	/	*E. coli*: 58.7	[44]
FN-GOQD/Ag	65.8	/	77.4	*E. coli*: 99.8<br>*B. subtilis*:97.3	[45]
HPEIGO/PES	153.5	77.4	51.7	*E. coli*:74.88	[46]

GO-pPES	50	/	80	*E. coli*:80	[47]
rGO-DDA	89.6	100（BSA）	89.1	*E. coli*:83.6	[48]
PSF/GO	158	/	/	*E. coli*:66	[49]
PES/CRGO-Ly	372.3	/	/	*E. coli*:71	[50]
PES/ZGO-NH	95.49	95（BSA）	84.4	*E. coli*:81.1<br>*B. subtilis*:85.7	[51]
